# Monoclonal Antibodies to the Thyrotropin Receptor

**DOI:** 10.1080/17402520500078238

**Published:** 2005-06

**Authors:** Takao Ando, Terry F. Davies

**Affiliations:** Department of Medicine Mount Sinai School of Medicine Box 1055, 1 Gustave L. Levy Place New York NY 10029 USA

## Abstract

The thyrotropin receptor (TSHR) is a seven transmembrane G-protein linked glycoprotein expressed on the thyroid cell surface and which, under the regulation of TSH, controls the production and secretion of thyroid hormone from the thyroid gland. This membrane protein is also a major target antigen in the autoimmune thyroid diseases. In Graves' disease, autoantibodies to the TSHR (TSHR-Abs) stimulate the TSHR to produce thyroid hormone excessively. In autoimmune thyroid failure, some patients exhibit TSHR-Abs which block TSH action on the receptor. There have been many attempts to generate human stimulating TSHR-mAbs, but to date, only one pathologically relevant human stimulating TSHR-mAb has been isolated. Most mAbs to the TSHR have been derived from rodents immunized with TSHR antigen from bacteria or insect cells. These antigens lacked the native conformation of the TSHR and the resulting mAbs were exclusively blocking or neutral TSHR-mAbs. However, mAbs raised against intact native TSHR antigen have included stimulating mAbs. One such stimulating mAb has demonstrated a number of differences in its regulation of TSHR post-translational processing. These differences are likely to be reflective of TSHR-Abs seen in Graves' disease.


*Clinical & Developmental Immunology*, June 2005; 12(2): 137–143

Taylor & Francis

Taylor & Francis Group

### Monoclonal antibodies to the thyrotropin receptor

TAKAO ANDO, & TERRY F. DAVIES

*Department of Medicine, Mount Sinai School of Medicine, Box 1055, 1 Gustave L. Levy Place, New York, NY 10029 USA*

**Abstract**
The thyrotropin receptor (TSHR) is a seven transmembrane G-protein linked glycoprotein expressed on the thyroid cell surface and which, under the regulation of TSH, controls the production and secretion of thyroid hormone from the thyroid gland. This membrane protein is also a major target antigen in the autoimmune thyroid diseases. In Graves' disease, autoantibodies to the TSHR (TSHR-Abs) stimulate the TSHR to produce thyroid hormone excessively. In autoimmune thyroid failure, some patients exhibit TSHR-Abs which block TSH action on the receptor. There have been many attempts to generate human stimulating TSHR-mAbs, but to date, only one pathologically relevant human stimulating TSHR-mAb has been isolated. Most mAbs to the TSHR have been derived from rodents immunized with TSHR antigen from bacteria or insect cells. These antigens lacked the native conformation of the TSHR and the resulting mAbs were exclusively blocking or neutral TSHR-mAbs. However, mAbs raised against intact native TSHR antigen have included stimulating mAbs. One such stimulating mAb has demonstrated a number of differences in its regulation of TSHR post-translational processing. These differences are likely to be reflective of TSHR-Abs seen in Graves' disease.

**Keywords:** *Autoantibodies, autoimmunity, epitope, Graves' disease*

### Thyrotropin receptor

The thyrotropin receptor (TSHR) is a G protein-coupled receptor with a large extracellular domain. This molecule is expressed on the surface of thyroid follicular epithelial cells to regulate thyroid growth, hormone production and release (Rapoport et al. 1998, Rees Smith et al. 1988, Davies et al. 2002). A TSHR holoreceptor is expressed on the plasma membrane (Misrahi et al. 1994, Tanaka et al. 1999) and subsequently undergoes intramolecular cleavage, resulting in the loss of an intervening region (TSHR residues ~316–366). This results in a two subunit structure, with α (or A) and β (or B) subunits held together by disulfide bonds (Rapoport et al. 1998). The conformational structure made up of the extracellular region of the receptor is the site of TSH binding. The extracellular α subunit (residues to ~316 after intra-molecular cleavages) is shed from the plasma membrane *in vitro* (Couet et al. 1996) and presumed to undergo the same process *in vivo.* The seven transmembrane regions and cytoplasmic β subunit (residues from ~366) of the receptor are involved in signal transduction and are detected in greater concentrations than the α subunits (Loosfelt et al. 1992, Tanaka et al. 1999) due to the α subunit shedding. In addition to this post-translational processing, the TSHR has also been shown to undergo homomeric multimer formation *in vivo* (Graves et al. 1996) and *in vitro* (Latif et al. 2001) and the TSH is able to monomerize these multimeric TSHR complexes (Latif et al. 2002).

### The TSHR and thyroid autoimmunity

The TSHR is a target antigen of T cells and autoantibodies in autoimmune thyroid disease. These diseases include hyperthyroid Graves' disease (GD) and hypothyroid Hashimoto's thyroiditis (HT). Autoantibodies to the TSHR (TSHR-Abs) may activate the TSHR, resulting in overproduction of thyroid hormone, or block the thyrotropic action of TSH, inducing thyroid atrophy and hypothyroidism (Rees Smith et al. 1988, Davies et al. 2002). In addition to these two classes of TSHR-Abs, there

Correspondence: T. F. Davies, Mount Sinai School of Medicine, Box 1055, 1 Gustave L. Levy Place, New York, NY 10029, USA. Tel: 1 212 241 4129. Fax: 1 212 241 4218. E-mail: terry.davies@mssm.edu

ISSN 1740-2522 print/ISSN 1740-2530 online © 2005 Taylor & Francis Group Ltd
DOI: 10.1080/17402520500078238

is a third class of TSHR-Abs called neutral antibodies, which bind to the TSHR without influencing TSH binding to the TSHR or TSH action. The presence of neutral TSHR antibodies was first reported in a patient with GD and monoclonal gammopathy (Tonacchera et al. 1996). Using affinity purified TSHR, it has now been suggested that sera from healthy normal controls may also contain neutral TSHR-Abs (Atger et al. 1999). However, the presence of these antibodies in healthy individuals seems to be very rare (Metcalfe et al. 2002) and the clinical significance of neutral antibodies to the TSHR is presently uncertain.

### TSHR autoantibodies

TSHR-Abs detected in the sera from patients with autoimmune thyroid disease or animals immunized with TSHR antigen are produced from peripheral, splenic and infiltrating intrathyroidal B cells (Table I). If a repertoire of TSHR monoclonal antibodies (mAbs) is to be generated, it should reflect the antibody repertoire which is present in the patients or immunized animals. Therefore, it is important to know the characteristics of serum TSHR-Abs as a reflection of the B cell repertoire from which they arise.

TSHR-Abs in sera from patients with GD have been shown to be oligoclonal IgG as determined by IgH (Zakarija 1983, Weetman et al. 1990) and IgL (Weetman et al. 1990) isotyping. Patient serum is thought to be often a mixture of stimulating and blocking antibodies (Kim et al. 2000, Minich et al. 2004) and probably also includes neutral TSHR antibodies (Tonacchera et al. 1996, Jaume et al. 1997). Many of these antibodies are detected by their ability to displace labeled TSH binding to porcine (Rees Smith et al. 1988) or human TSHRs (Costagliola et al. 1999). This activity can be found in nearly 100% of untreated GD patients (Costagliola et al. 1999, Maugendre and Massart 2001, Schott et al. 2004) and, in general, decreases with treatment of the disease (Maugendre and Massart 2001, Schott et al. 2004). This TSH competing activity, by definition, is a reflection of both stimulating and blocking antibodies although in GD most such activity is a reflection of stimulators (Ando and Davies 2005). Stimulating antibodies induce cAMP production as a result of TSHR activation while blocking TSHR antibodies inhibit cAMP generation induced by TSH; thus allowing for their easy distinction. TSHR-Abs are present in very low concentrations in the serum of patients (< 10 ug/ml) (Jaume et al. 1997, Chazenbalk et al. 1997, Atger et al. 1999, Cornelis et al. 2001) yet, in GD, are still able to induce hyperthyroidism. Therefore, pathophysiologically relevant monoclonal stimulating and blocking antibodies should show TSH competing activity, and induce or inhibit cAMP production in low concentrations.

### Factors affecting the generation of rodent mAbs to the TSHR

To obtain mAbs in a rodent, careful consideration of the nature of the antigen is mandatory. There is a reduced chance of inducing antibody to a conformational epitope if it is not present in the antigen used for immunization. In order to induce stimulating TSHR-Abs by immunization, it has been found that the TSHR antigen must be intact and not denatured or fragmented. However, in order to induce TSHR-Abs to short residues (linear epitope), denatured TSHR antigen without its intact conformation has been successful.

Similarly, the type of mAbs obtained, either human or rodent, will depend on their detection by the screening method used (Table II). As reviewed in Ando and Davies (2005), stimulating antibodies recognize conformational epitopes on the α subunit of the TSHR. Blocking antibodies recognize either the same or closely related conformational binding sites that stimulating antibodies bind to, or they recognize linear epitopes including those on the N terminus of the β subunit of the TSHR. Therefore, if the TSHR antigen used for screening does not have intact conformation, then stimulating antibodies and some

Table I. TSHR antigens and mAb generation.

| B cell | Transformation | TSHR antigen | Cons and pros |
|---|---|---|---|
| Human PBMC* | Mouse plasmacytoma<br>Epstein bar virus | Native TSHR *in vivo*[†]<br>Native TSHR *in vivo* | Stimulating mAbs could potentially be obtained, but clones tend to be unstable. |
| Animal SPC* | Mouse myeloma | Denatured TSHR | mAbs often recognize only the denatured antigen. |
| | | Native TSHR | Antigen may not be intact at the site of immunization. |
| | | Native TSHR *in situ*[‡] | mAbs tend to recognize only the native TSHR. |

*PBMC— peripheral blood mononuclear cells; SPC—spleen cells.
[†]TSHR expressed on the thyroid gland.
[‡]TSHR expressed by an expression vector at the site of immunization.

Table II. mAb screening.

| Method | TSHR antigen | Pros | Cons |
|---|---|---|---|
| ELISA | Denatured/native | Ideal for mass screening. Not expensive. | May not detect mAbs to native conformation of the TSHR |
| Labeled TSH* | Solubilized/native | Detected only stimulating and blocking mAbs | Miss neutral mAbs. Costly and labor intensive. Radioisotope used.† |
| Camp | Native form | Detects only stimulating and blocking mAbs. | Miss neutral mAbs. Needs cell culture. Costly and labor intensive. Radioisotope used† |
| FACS | Native form | Best to detect mAbs to the native TSHR. Not expensive | Needs cell culture and labor intensive. May miss mAbs to the linear epitopes. |

*Labeled TSH indicates TSH binding competition assay and when this assay is performed with the native TSHR, cell culture is needed. TSHR conformation is different between the solubilized and the native TSHR.
†Non-radioisotope kits are commercially available.

blocking antibodies, will not be detected. Similarly, one may be able to obtain mAbs which only react with denatured TSHR when screening is dependent on using denatured TSHR antigen. Hence, the screening method is the second important factor in addition to the immunizing antigen.

### Monoclonal antibodies to the TSHR

*Use of peripheral B cells from GD patients*

*Human–mouse hybridoma formation*. The first human TSHR-mAbs in the literature appeared in Valente et al. (1982) and were generated by using mouse myeloma cells to fuse and transform target lymphocytes from patients with GD resulting in heterohybridomas. Subsequently, human myeloma cells were also used to generate mAbs (Yoshida et al. 1988). More recently, TSHR-mAbs were produced from a patient with HT by using a mouse myeloma partner (Kohn et al. 1997). These mAbs, mostly IgG, showed stimulating or blocking activity only at high concentrations (>100 ug/ml). Binding of some of these stimulating mAbs to thyroid membrane was significantly inhibited by TSH, but these mAbs did not show any TSH competing activity themselves. Such studies clearly indicated that the human TSHR mAbs described, and which required >100 ug/ml to see activity, were of low affinity and not likely to be present in high enough concentrations in the serum of patients. Therefore, no clinical significance could be assigned to them.

*EBV transformation of B cells*. Epstein bar virus (EBV) infects and transforms human B cells. This ability has been utilized to immortalize Ab-producing B cells from peripheral blood lymphocytes. Baker et al. (1988) reported the result to be an IgM TSHR mAb by using this method. This IgM mAb showed no TSH competition but blocked TSH activity. It reacted with 15–18 kDa products on Western blots, but the detected bands did not correspond to the molecular weight of the TSHR, suggesting that its blocking activity was non-specific. More recently, after eliminating IgM bearing peripheral B cells by negative-sorting, IgG mAbs with weak TSHR stimulating activity (up to 3 fold stimulation of cAMP) were isolated (Li et al. 1995). Although the poor stimulating activity of these mAbs was secondary to low affinity binding, one was used to generate a transgenic mouse model of GD (Kim-Saijo et al. 2003). This mAb (B6B7) was originally reported as IgG, but the transgenic animals carried an IgM version of this mAb due to a class switch. There was detectable (~50 ug/ml) human IgM in the mouse serum and in some animals this transgene product increased serum T4 levels. However, the maximum increase observed was a doubling in the average level of serum thyroxine when compared to control mice. Furthermore, ~40% of the transgenic mice did not show any increase in T4 levels even with detectable human IgM in the serum. Nevertheless, despite the low potency of the TSHR mAb some of these mice also showed thyroid hypertrophy (Kim-Saijo et al. 2003) and this remains an interesting model to be verified by other investigators. Recently, a potent stimulating human IgG TSHR mAb has been successfully isolated using this technique after screening >16,000 wells by a TSH binding competition assay (Sanders et al. 2003). This mAb generated from a patient with GD showed potent thyroid stimulating activity at low concentrations (~2–3 times cAMP stimulation at ~1 ng/ml), much stronger than previous human mAbs. Hence, human mAbs can be obtained from patients with GD but the difficulty in selecting them is consistent with the low levels of TSHR-Abs present (Chazenbalk et al. 1997, Jaume et al. 1997, Atger et al. 1999, Cornelis et al. 2001) and a low frequency TSHR-Ab secreting plasma cells.

*TSHR-mAbs from animals immunized with TSHR antigen*

*mAbs raised against recombinant denatured TSHR antigen*. Loosfelt et al. (1992) generated several mAbs raised against the TSHR ectodomain and transmembrane region of the TSHR. These mAbs confirmed the two subunit structure of the TSHR as originally shown using radio labeled TSH cross-linking (Kajita et al. 1985). These probes also demonstrated β subunit predominance (Loosfelt et al. 1992) which has been confirmed in other laboratories (Tanaka et al. 1999). Using two mAbs raised against TSHR α subunit residues 19–243 but with distinct epitopes, this group further detected shed TSHR α subunits in the cell culture medium (Couet et al. 1996). Such reactivity was not detected with mAbs to the intracellular tail. However, the TSH competing activity of the mAbs in these studies has not been reported. Seeraramaiah et al. (1995) immunized mice with insect-derived TSHR ectodomain and generated 23 mAbs, including 2 mAbs with TSH competing and blocking activity. However, this activity was seen only in high concentrations (>250 ug/ml). These mAbs had linear epitopes within TSHR residues 277–296. Nicholson et al. (1996) generated 5 mAbs raised against TSHR ectodomain produced in *E. coli* and insect cells. Some of the mAbs in this study have been widely used for studies of the TSHR. For example, their A9 mAb recognized TSHR residues 147–229, the native TSHR expressed in thyroid tissue and frozen specimens (Nicholson et al. 1996) and the TSHR in immunoprecipitation studies (Tanaka et al. 1999, Chazenbalk et al. 2002). Their mAb A10 recognized residues 22–35 (Nicholson et al. 1996) and has been especially useful in immunoblot studies (Vlase et al. 1997). Davies et al. (1998) also generated 10 mAbs raised against mouse TSHR ectodomain produced in insect cells. These mAbs, when studied in an ELISA format, showed preferential recognition of mouse TSHRs over human TSHRs. Certain mAbs showed TSH competing and blocking activity, but again only at relatively high concentrations (>50 ug/ml).

Most of the above mentioned mAbs were generated and screened for using the same TSHR antigen. The mAbs reacted well with the antigen used for immunization but did not react (Seeraramaiah et al. 1995, Nicholson et al. 1996, Davies et al. 1998), or reacted much less (Seeraramaiah et al. 1995, Davies et al. 1998) with the native TSHR. However, other studies have used a more logical approach; immunizing with the available denatured antigen but screening with the native TSHR antigen. Johnstone et al generated mAb 2C11 raised against the TSHR ectodomain generated in bacteria (Johnstone et al. 1994). This antibody was obtained by screening with CHO cells expressing the native TSHR, and it reacted with a linear epitope of residues 355–358 (Shepherd et al. 1999). More mAbs were later generated by this method but all are bound to TSHR residues 381–384 and blocked rather than stimulated TSH induced cAMP generation (~80% blockade at ~1 ug/ml). Oda et al. (1998) took a different approach to screening by using immunoprecipitation of radio-labeled *in vitro* translated TSHR but this TSHR preparation was shown not to be recognized by TSHR-Abs present in GD sera (Prentice et al. 1997). One antibody (3C7), with a linear epitope to residues 246–260, showed TSH competing activity (>20%) but, once again, only observed with more than ~30 ug/ml and did not recognize native TSHR expressed on CHO cells (Oda et al. 1998).

Hence, mAbs screened by using native TSHR preparations (Johnstone et al. 1994, Shepherd et al. 1999) but with linear epitopes were able to interact with the native TSHR. One mAb recognizing TSHR residues 381–384 showed strong (~80%) blocking activity at 1 ug/ml (Shepherd et al. 1999), which is pathologically relevant. However, none of the mAbs had thyroid stimulating activity.

*mAbs raised to the native TSHR*. Yavin et al. (1981) claimed the *first* murine mAbs against the TSHR after immunizing with solubilized thyroid membrane preparations. While these mAbs may have competed for labeled TSH binding they were not characterized as to their concentration or specificity and appeared to be against a glycoprotein "component" of the receptor. Subsequently, Marion et al. (1992) immunized mice with a purified TSHR preparation derived from an immortalized thyroid cell hybridoma (GEJ) (Karsenty et al. 1985). Among the mAbs generated, mAb 34A showed binding to TSHRs expressed on both GEJ and CHO cells and inhibited labeled TSH binding to such TSHRs. It was also claimed that 34A stimulated cAMP production (Marion et al. 1992). Using affinity purified TSHR from CHO-TSHR cells Oda et al generated >40 mAbs to the TSHR (Oda et al. 2000). Although they used the purified native TSHR as antigen, all the mAbs bound denatured TSHR produced in bacteria. However, one mAb (8E3) to residues 381–385 was active against the intact TSHR in relatively low concentrations (5 ug/ml) as evidenced by strong (~70%) TSH competing activity. In these studies, none of the mice became hyperthyroid and no convincing stimulating mAbs were generated by these native TSHR immunizations.

*mAbs raised against the native TSHR* in situ. While Shimojo et al. (1996) were the first to describe a murine model of hyperthyroid GD, based on immunization with fibroblasts expressing the TSHR, no mAbs to the TSHR were produced by this approach. Costagliola et al. (1998) used genetic immunization of Balb/c mice with human TSHR cDNA and induced TSHR-Abs interacting with the native TSHR, including evidence for the presence of stimulating TSHR-Abs in their sera. However, the animals remained euthyroid (Costagliola et al. 1998).

By FACS screening, they generated neutral mAbs with conformational determinants. One of these mAbs was utilized to fix the TSHR to a solid phase in second generation TSH binding competition assay (Costagliola et al. 1999). An additional blocking mAb was generated to residues around 382–383 (Costagliola et al. 2002b), which was used to identify sulfated tyrosine residues involved in TSH–TSHR interaction (Costagliola et al. 2002b). Subsequently, these investigators used triple screening of their hybridomas by FACS, TSH binding competition and cAMP generation assays and found one IgG mAb with TSHR stimulating activity (Costagliola et al. 2002a). However, this mAb had an activity that stimulated cAMP generation by 2–3 times basal at a concentration of $\sim$300 ng/ml (Costagliola et al. 2002a). Using this same genetic immunization approach and conventional purified TSHR immunization, Jeffreys et al. (2002) produced $>$130 TSHR blocking and neutral mAbs, including many with TSH competing activity, and used this panel to locate residues 246–260, 277–296 and 381–385 as the sites involved in a TSH binding pocket.

By using a TSHR cDNA immunization protocol in outbred mice first reported by Costagliola et al. (2000), Sanders et al. successfully generated three stimulating mAbs by screening with TSH competition assays from those mice which developed TSH competing activity in their sera (Sanders et al. 2002). These three mAbs had different binding affinities to the TSHR and different coding sequences for their variable regions. Significant stimulation (more than $2\times$ increases in basal cAMP) was observed at $\sim$20 ng/ml for the most potent mAbs and at 2 ug/ml for the least potent mAb. Importantly, these three stimulating mAbs showed binding competition with each other and also competed with TSHR-Abs from patients with GD (Costagliola et al. 1998). This study indicated that the epitopes recognized by stimulating TSHR-Abs in patients were similar to those recognized by the stimulating mAbs raised in mice and were not distributed heterogeneously on the TSHR $\alpha$ subunit.

A mouse model of GD was also induced by an adenovirus vector expressing the human TSHR (Nagayama et al. 2002) and this was reproduced in the hamster (Ando et al. 2003). By screening with FACS assays, a panel of hamster TSHR-mAbs were generated including a stimulating mAb (MS-1) which increased cAMP production 2 fold with $<$20 ng/ml IgG (Ando et al. 2002). Among these mAbs, both stimulating and blocking varieties showed conformational recognition of the TSHR $\alpha$ subunit (Ando et al. 2002), but these two epitopes were distinct (Ando et al. 2004b). Epitope analysis of this panel of hamster mAbs showed a restricted epitope distribution on the TSHR and this was compatible with the TSHR-Ab repertoire present in the originating hamster sera (Ando et al. 2004a). Three mouse stimulating mAbs (Sanders et al. 2002) shared the same conformational epitope on the $\alpha$ subunit of the TSHR as a hamster stimulating mAb (Ando et al. 2004a).

### Bioactivity of stimulating TSHR mAbs

Among the stimulating mAbs generated to date, only one TSHR-mAb (MS-1) has been examined for its *in vivo* bioactivity. When injected into mice, MS-1 induced a marked increase in serum thyroid hormones (Ando et al. 2004b) but mice which were chronically exposed to oversaturating concentrations of MS-1, showed a diminution in thyroid hormone output most likely, secondary to TSHR desensitization and down regulation as demonstrated *in vitro* (Ando et al. 2004b). Hence, this TSHR-mAb induced concentration dependent regulation of TSHR function. Such data may explain the poor correlation between thyroid function and serum titers of stimulating antibodies in GD.

The influence of MS-1 on post-translational processing of the TSHR has also been studied. There are constitutive multimeric TSHR complexes in the cell membrane and TSH is able to induce their monomerization (Latif et al. 2002). TSH is also able to enhance intra-molecular cleavage of the TSHR when assessed by a flow cytometric cleavage assay (Ando et al. 2002). However, MS-1 stimulation failed to induce TSHR monomer formation as measured by the speed of lateral movement of TSHRs after stimulation (Latif et al. 2004) and failed to enhance cleavage (Ando et al. 2002). These differences may have been due to the bivalent nature of MS-1 IgG since MS-1 Fab was able to act like TSH and increase the lateral movement of TSHRs and able to accelerate TSHR cleavage (Latif et al. 2004). These data indicate significant differences in the regulation of the TSHR by TSH compared to TSHR-Abs (Table III) and may help to explain the sustained and prolonged TSHR stimulation seen in patients with GD.

### Summary

There have been many reports of human TSHR-mAbs in the literature. Only recently, mAbs which interact with the native TSHR with high affinity have been generated. These mAbs may block or stimulate the thyroid glands or may be neutral in their activity and reveal insights into the antibody repertoire in autoimmune thyroid disease. Since human TSHR-mAbs have been difficult to isolate, mAbs from animal models will continue to be generated as tools to study the molecular biology of the TSHR. In addition, TSHR-mAbs have important diagnostic and therapeutic potential.

Table III. Regulation of TSHR by TSH and a stimulating TSHR mAb (MS-1).

| | TSH | MS-1 IgG | MS-1 Fab | References |
|---|---|---|---|---|
| Activation* | Yes | Yes | Yes | Ando et al. (2002), Latif et al. (2004) |
| Down regulation† | Yes | Yes | ND‡ | Ando et al. (2004b), Latif et al. (2004) |
| Desensitization | Yes | Yes | ND | Ando et al. (2004b) |
| Intra-molecular cleavage | Yes | No | Yes | Ando et al. (2002), Latif et al. (2004) |
| Shedding¶ | Yes | ND | ND | Latif et al. (2004) |
| Monomerization§ | Yes | No | Yes | Latif et al. (2002), Latif et al. (2004) |

* Activation of TSHR was determined by increase of the intracellular cAMP.
† Down regulation was measured by loss of surface TSHRs using TSHR-mAbs.
‡ ND indicates not done.
¶ Shedding was studied in N-terminus tagged TSHR.
§ Monomerization was studied by the lateral movement of green fluorescent protein (GFP) tagged TSHR at the C terminus of the TSHR stably expressed on CHO cells.

### Acknowledgements

Supported in part by NIH grants DK52464, DK45011 and AI24671 (to TFD), and the David Owen Segal Endowment (to TA).

### References

Ando T, Davies TF. 2005. Thyrotropin receptor antibodies: New insights into their actions and clinical relevance. Best Pract Res Clin Endocrinol Metab, in press.

Ando T, Latif R, Pritsker A, Moran T, Nagayama Y, Davies TF. 2002. A monoclonal thyroid-stimulating antibody. J Clin Investig 110:1667–1674.

Ando T, Imaizumi M, Graves P, Unger P, Davies TF. 2003. Induction of thyroid-stimulating hormone receptor autoimmunity in hamsters. Endocrinology 144:671–680.

Ando T, Latif R, Daniel S, Eguchi K, Davies TF. 2004. Dissecting linear and conformational epitopes on the native thyrotropin receptor. Endocrinology, in press.

Ando T, Latif R, Davies TF. 2004. Concentration-dependent regulation of the thyrotropin receptor function by thyroid-stimulating antibody. J Clin Investig 113:1589–1595.

Atger M, Misrahi M, Young J, et al. 1999. Autoantibodies interacting with purified native thyrotropin receptor. Eur J Biochem 265:1022–1031.

Baker Jr, JR, Saunders NB, Kaulfersch W, Burman KD. 1988. Development of a human monoclonal antibody from a Graves' disease patient that identifies a novel thyroid membrane antigen. J Immunol 140:2593–2599.

Chazenbalk GD, Jaume JC, McLachlan SM, Rapoport B. 1997. Engineering the human thyrotropin receptor ectodomain from a non- secreted form to a secreted, highly immunoreactive glycoprotein that neutralizes autoantibodies in Graves' patients' sera. J Biol Chem 272:18959–18965.

Chazenbalk GD, Pichurin P, Chen CR, et al. 2002. Thyroid-stimulating autoantibodies in Graves disease preferentially recognize the free A subunit, not the thyrotropin holoreceptor. J Clin Investig 110:209–217.

Cornelis S, Uttenweiler-Joseph S, Panneels V, Vassart G, Costagliola S. 2001. Purification and characterization of a soluble bioactive amino-terminal extracellular domain of the human thyrotropin receptor. Biochemistry 40:9860–9869.

Costagliola S, Rodien P, Many MC, Ludgate M, Vassart G. 1998. Genetic immunization against the human thyrotropin receptor causes thyroiditis and allows production of monoclonal antibodies recognizing the native receptor. J Immunol 160:1458–1465.

Costagliola S, Morgenthaler NG, Hoermann R, et al. 1999. Second generation assay for thyrotropin receptor antibodies has superior diagnostic sensitivity for Graves' disease. J Clin Endocrinol Metab 84:90–97.

Costagliola S, Many MC, Denef JF, Pohlenz J, Refetoff S, Vassart G. 2000. Genetic immunization of outbred mice with thyrotropin receptor cDNA provides a model of Graves' disease. J Clin Investig 105:803–811.

Costagliola S, Franssen JD, Bonomi M, et al. 2002. Generation of a mouse monoclonal TSH receptor antibody with stimulating activity. Biochem Biophys Res Commun 299:891–896.

Costagliola S, Panneels V, Bonomi M, et al. 2002. Tyrosine sulfation is required for agonist recognition by glycoprotein hormone receptors. EMBO J 21:504–513.

Couet J, Sar S, Jolivet A, Hai MT, Milgrom E, Misrahi M. 1996. Shedding of human thyrotropin receptor ectodomain. Involvement of a matrix metalloprotease. J Biol Chem 271:4545–4552.

Davies TF, Bobovnikova Y, Weiss M, Vlase H, Moran T, Graves PN. 1998. Development and characterization of monoclonal antibodies specific for the murine thyrotropin receptor. Thyroid 8:693–701.

Davies TF, Marians R, Latif R. 2002. The TSH receptor reveals itself. J Clin Investig 110:161–164.

Graves PN, Vlase H, Bobovnikova Y, Davies TF. 1996. Multimeric complex formation by the thyrotropin receptor in solubilized thyroid membranes. Endocrinology 137:3915–3920.

Jaume JC, Kakinuma A, Chazenbalk GD, Rapoport B, McLachlan SM. 1997. Thyrotropin receptor autoantibodies in serum are present at much lower levels than thyroid peroxidase autoantibodies: Analysis by flow cytometry. J Clin Endocrinol Metab 82:500–507.

Jeffreys J, Depraetere H, Sanders J, et al. 2002. Characterization of the thyrotropin binding pocket. Thyroid 12:1051–1061.

Johnstone AP, Cridland JC, Da Costa CR, Harfst H, Shepherd PS. 1994. Monoclonal antibodies that recognize the native human thyrotropin receptor. Mol Cell Endocrinol 105:R1–R9.

Kajita Y, Rickards CR, Buckland PR, Howells RD, Rees Smith B. 1985. Analysis of thyrotropin receptors by photoaffinity labelling. Orientation of receptor subunits in the cell membrane. Biochem J 227:413–420.

Karsenty G, Michel-Bechet M, Charreire J. 1985. Monoclonal human thyroid cell line GEJ expressing human thyrotropin receptors. Proc Natl Acad Sci USA 82:2120–2124.

Kim WB, Chung HK, Park YJ, Tahara K, Kohn LD, Cho BY. 2000. The prevalence and clinical significance of blocking thyrotropin receptor antibodies in untreated hyperthyroid Graves' disease. Thyroid 10:579–586.

Kim-Saijo M, Akamizu T, Ikuta K, et al. 2003. Generation of a transgenic animal model of hyperthyroid Graves' disease. Eur J Immunol 33:2531–2538.

Kohn LD, Suzuki K, Hoffman WH, et al. 1997. Characterization of monoclonal thyroid-stimulating and thyrotropin binding-inhibiting autoantibodies from a Hashimoto's patient whose children had intrauterine and neonatal thyroid disease. J Clin Endocrinol Metab 82:3998–4009.

Latif R, Graves P, Davies TF. 2001. Oligomerization of the human thyrotropin receptor: Fluorescent protein- tagged hTSHR reveals post-translational complexes. J Biol Chem 276:45217–45224.

Latif R, Graves P, Davies TF. 2002. Ligand-dependent inhibition of oligomerization at the human thyrotropin receptor. J Biol Chem 277:45059–45067.

Latif R, Ando T, Davies TF. 2004. Monomerization as a prerequisite for intramolcular cleavage and shedding of the thyrotropin receptor. Endocrinology, in press.

Li H, Akamizu T, Okuda J, et al. 1995. Isolation of Epstein-Barr-virus-transformed lymphocytes producing IgG class monoclonal antibodies using a magnetic cell separator (MACS): Preparation of thyroid-stimulating IgG antibodies from patients with Graves' disease. Biochem Biophys Res Commun 207:985–993.

Loosfelt H, Pichon C, Jolivet A, et al. 1992. Two-subunit structure of the human thyrotropin receptor. Proc Natl Acad Sci USA 89:3765–3769.

Marion S, Ropars A, Ludgate M, Braun JM, Charreire J. 1992. Characterization of monoclonal antibodies to the human thyrotropin receptor. Endocrinology 130:967–975.

Maugendre D, Massart C. 2001. Clinical value of a new TSH binding inhibitory activity assay using human TSH receptors in the follow-up of antithyroid drug treated Graves' disease. Comparison with thyroid stimulating antibody bioassay. Clin Endocrinol (Oxf) 54:89–96.

Metcalfe R, Jordan N, Watson P, et al. 2002. Demonstration of immunoglobulin G, A, and E autoantibodies to the human thyrotropin receptor using flow cytometry. J Clin Endocrinol Metab 87:1754–1761.

Minich WB, Lenzner C, Morgenthaler NG. 2004. Antibodies to TSH-receptor in thyroid autoimmune disease interact with monoclonal antibodies whose epitopes are broadly distributed on the receptor. Clin Exp Immunol 136:129–136.

Misrahi M, Ghinea N, Sar S, et al. 1994. Processing of the precursors of the human thyroid-stimulating hormone receptor in various eukaryotic cells (human thyrocytes, transfected L cells and baculovirus-infected insect cells). Eur J Biochem 222:711–719.

Nagayama Y, Kita-Furuyama M, Ando T, et al. 2002. A novel murine model of Graves' hyperthyroidism with intramuscular injection of adenovirus expressing the thyrotropin receptor. J Immunol 168:2789–2794.

Nicholson LB, Vlase H, Graves P, et al. 1996. Monoclonal antibodies to the human TSH receptor: Epitope mapping and binding to the native receptor on the basolateral plasma membrane of thyroid follicular cells. J Mol Endocrinol 16:159–170.

Oda Y, Sanders J, Roberts S, et al. 1998. Binding characteristics of antibodies to the TSH receptor. J Mol Endocrinol 20:233–244.

Oda Y, Sanders J, Evans M, et al. 2000. Epitope analysis of the human thyrotropin (TSH) receptor using monoclonal antibodies. Thyroid 10:1051–1059.

Prentice L, Sanders J, Perez M, et al. 1997. Thyrotropin (TSH) receptor autoantibodies do not appear to bind to the TSH receptor produced in an *in vitro* transcription/translation system. J Clin Endocrinol Metab 82:1288–1292.

Rapoport B, Chazenbalk GD, Jaume JC, McLachlan SM. 1998. The thyrotropin (TSH) receptor: Interaction with TSH and autoantibodies. Endocr Rev 19:673–716.

Rees Smith B, McLachlan SM, Furmaniak J. 1988. Autoantibodies to the thyrotropin receptor. Endocr Rev 9:106–121.

Sanders J, Jeffreys J, Depraetere H, et al. 2002. Thyroid-stimulating monoclonal antibodies. Thyroid 12:1043–1050.

Sanders J, Evans M, Premawardhana LD, et al. 2003. Human monoclonal thyroid stimulating autoantibody. Lancet 362:126–128.

Schott M, Morgenthaler NG, Fritzen R, et al. 2004. Levels of autoantibodies against human TSH receptor predict relapse of hyperthyroidism in Graves' disease. Horm Metab Res 36:92–96.

Seeraramaiah GS, Wagle NM, Morris JC, Prabhakar BS. 1995. Generation and characterization of monoclonal antibodies to the human thyrotropin (TSH) receptor: Antibodies can bind to discrete conformational or linear epitopes and block TSH binding. Endocrinology 136:2817–2824.

Shepherd PS, Da Costa CR, Cridland JC, Glimore KS, Johnstone AP. 1999. Identification of an important thyrotropin binding sites on the human thyrotropin receptor using monoclonal antibodies. Mol Cell Endocrinol 149:197–206.

Shimojo N, Kohno Y, Yamaguchi K, et al. 1996. Induction of Graves-like disease in mice by immunization with fibroblasts transfected with the thyrotropin receptor and a class II molecule. Proc Natl Acad Sci USA 93:11074–11079.

Tanaka K, Chazenbalk GD, McLachlan SM, Rapoport B. 1999. Subunit structure of thyrotropin receptors expressed on the cell surface. J Biol Chem 274:33979–33984.

Tonacchera M, Costagliola S, Cetani F, et al. 1996. Patient with monoclonal gammopathy, thyrotoxicosis, pretibial myxedema and thyroid-associated ophthalmopathy; demonstration of direct binding of autoantibodies to the thyrotropin receptor. Eur J Endocrinol 134:97–103.

Valente WA, Vitti P, Yavin Z, et al. 1982. Monoclonal antibodies to the thyrotropin receptor: Stimulating and blocking antibodies derived from the lymphocytes of patients with Graves disease. Proc Natl Acad Sci USA 79:6680–6684.

Vlase H, Matsuoka N, Graves PN, Magnusson RP, Davies TF. 1997. Folding-dependent binding of thyrotropin (TSH) and TSH receptor autoantibodies to the murine TSH receptor ectodomain. Endocrinology 138:1658–1666.

Weetman AP, Yateman ME, Ealey PA, et al. 1990. Thyroid-stimulating antibody activity between different immunoglobulin G subclasses. J Clin Investig 86:723–727.

Yavin E, Yavin Z, Schneider MD, Kohn LD. 1981. Monoclonal antibodies to the thyrotropin receptor: implications for receptor structure and the action of autoantibodies in Graves disease. Proc Natl Acad Sci USA 78:3180–3184.

Yoshida T, Ichikawa Y, Ito K, Homma M. 1988. Monoclonal antibodies to the thyrotropin receptor bind to a 56-kDa subunit of the thyrotropin receptor and show heterogeneous bioactivities. J Biol Chem 263:16341–16347.

Zakarija M. 1983. Immunochemical characterization of the thyroid-stimulating antibody (TSAb) of Graves' disease: Evidence for restricted heterogeneity. J Clin Lab Immunol 10:77–85.

